# Mapping the potential use of endectocide-treated cattle to reduce malaria transmission

**DOI:** 10.1038/s41598-019-42356-x

**Published:** 2019-04-09

**Authors:** Susan S. Imbahale, Julia Montaña Lopez, Joe Brew, Krijn Paaijmans, Cassidy Rist, Carlos Chaccour

**Affiliations:** 1 Department of Applied and Technical Biology, The Technical University of Kenya, Nairobi, Kenya; 2 ISGlobal, Hospital Clínic-Universitat de Barcelona, Barcelona, Spain; 3 Centro de Investigação em Saúde de Manhiça, Maputo, Mozambique; 4 Center for Evolution and Medicine, School of Life Sciences, Arizona State University, Tempe, AZ USA; 5 The Biodesign Center for Immunotherapy, Vaccines and Virotherapy, Arizona State University, Tempe, AZ USA; 6 Virginia-Maryland College of Veterinary Medicine, Virginia Tech, Blacksburg, VA USA; 7 Ifakara Health Institute, Ifakara, United Republic of Tanzania; 8 Facultad de Medicina, Universidad de Navarra, Pamplona, Spain

## Abstract

Treating cattle with endectocide is a longstanding veterinary practice to reduce the load of endo and ectoparasites, but has the potential to be added to the malaria control and elimination toolbox, as it also kills malaria mosquitoes feeding on the animals. Here we used openly available data to map the areas of the African continent where high malaria prevalence in 2–10 year old children coincides with a high density of cattle and high density of the partly zoophilic malaria vector *Anopheles arabiensis*. That is, mapping the areas where treating cattle with endectocide would potentially have the greatest impact on reducing malaria transmission. In regions of Africa that are not dominated by rainforest nor desert, the map shows a scatter of areas in several countries where this intervention shows potential, including central and eastern sub-Saharan Africa. The savanna region underneath the Sahel in West Africa appears as the climatic block that would benefit to the largest extent from this intervention, encompassing several countries. West Africa currently presents the highest under-10 malaria prevalence and elimination within the next twenty years cannot be contemplated there with currently available interventions alone, making the use of endectocide treated cattle as a complementary intervention highly appealing.

## Introduction

### Malaria control and elimination

Malaria continues to be one of the ten leading causes of death in low-income countries^1^, with 92% of all new malaria cases in 2017 being confined to the WHO African Region^2^. Six African countries (Nigeria, Democratic Republic of Congo, Burkina Faso, United Republic of Tanzania, Sierra Leone and Niger) accounted for almost half (49%) of all malaria deaths in 2017^2^. Remarkable advances in the fight against malaria had been achieved between the years 2000–2015, yet these stalled in 2015^3^. Despite countries setting the ambitious goal of reducing malaria case incidence by 40% in 2020, relative to 2015, under the WHO Global Technical Strategy for malaria 2016–30^4^, the world is far from reaching the goal since no significant progress has been made in reducing global malaria cases in the 2015–2017 period^2^. A number of challenges are hindering the attainment of this goal, including insufficient financing of malaria interventions, non-universal access to prevention and care, and the continued emergence of resistance to antimalarial medicines and insecticides^2^. In all major malaria vectors, resistance to the four main classes of insecticide (pyrethroids, organochlorines, carbamates and organophosphates) is widespread across malaria endemic regions^2^.

### Vector control as the pillar intervention against malaria

Targeting the mosquito vector has been and will remain one of the pillars of malaria control, elimination and eradication efforts^2,5^. The core vector control interventions (long-lasting insecticidal nets, or LLINs, and indoor residual spraying, or IRS) contributed to approximately 78% of the 663 million cases averted from 2000 to 2015^6^. These interventions have been highly effective as they reduce daily mosquito survival rates as well as rates of biting on humans, two parameters that drive onward malaria transmission^7^ due to their importance in determining vectorial capacity (the number of new infections induced by a given vector population)^8^. However, both LLINs and IRS interventions target anthropophagic, endophagic and endophilic mosquitoes, which are respectively vectors that are heavily reliant on human blood, feed predominantly indoors, and/or rest indoors^9^.

What LLINs and IRS interventions do not effectively address are mosquito behaviors that allow them to avoid contact with insecticides such as outdoor biting and resting, early exiting from houses after feeding, biting at dusk/dawn or at times when humans are not protected by nets and partial feeding upon livestock^10,11^. These behaviors create advantageous temporal and spatial gaps for the mosquito which contribute to residual malaria transmission, defined as “Persistence of malaria transmission following the implementation in time and space of a widely effective malaria programme”^8^. Controlling residual transmission requires new, complementary mosquito control strategies that go beyond the household walls^12,13^. One notable problematic vector species in Africa is *Anopheles arabiensis*, which can exhibit all of these behaviors to some degree, in particular when choosing hosts for bloodmeals. *An. arabiensis* collected at different sites display vast variability (from 0% to 80%) in the proportion of bloodmeals obtained from humans versus other animals^12^.

### The role of livestock in malaria transmission

Livestock play an important role in the epidemiology of several of the most important diseases of man by acting as reservoirs for zoonotic pathogens (e.g. zoonotic tuberculosis, brucellosis, etc.) that can be transmitted to humans via direct contact, aerosol, ingestion, fomites, or vector. However, the role of livestock as a source of blood-meal for arthropod vectors of non-zoonotic human diseases has also been shown to influence epidemiologic patterns of human diseases, including malaria^14^.

Human *Plasmodium* parasites are not infectious to livestock, but zooprophylaxis has long been proposed as a complementary strategy to reduce malaria transmission^15^. This is achieved when infected mosquitoes are diverted to alternative blood-sources that constitute “dead-end” hosts and prevent the amplification of the parasite^15^. One important pitfall in the concept of zooprophylaxis is that although human *Plasmodium* parasites cannot infect livestock, when animals are kept close to humans they can increase malaria transmission by attracting increasing numbers of mosquitoes in a phenomenon known as zoopotentiation^15,16^. Zoopotentiation has been shown to be exacerbated in areas with semi-intensive or semi-extensive cattle production, in which cattle graze freely during the day but sleep inside or in close proximity to human dwellings^17^. This type of production system is common practice in agro-pastoral and mixed crop livestock systems found throughout West Africa and in focal areas within East and Southern Africa^18^.

Although partially zoophilic vectors such as *An. arabiensis* feed upon animals and bite humans only occasionally and opportunistically^12^, feeding upon animals is often coupled with other resilient behaviors such as outdoor biting and avoidance of insecticide treated indoor surfaces. These resilient behaviors allow mosquitoes to thrive in the presence of good LLIN and/or IRS coverage^19^ and thus enable residual malaria transmission^11^. In many African countries, cattle are often dipped with insecticides to treat and prevent ectoparasites, a practice that has shown potential to control zoophagic vectors^13,14,20^ and thereby counteracting zoophagy-driven residual transmission. Dipping, however, has a relatively short duration against mosquitoes and is not free of safety issues caused by ingestion via licking. One attractive emerging alternative is the treatment of livestock with systemic insecticides or endectocides.

### Endectocides

Endectocides are a type of systemic insecticide that have activity against both endo-parasites and ecto-parasites and are commonly used in veterinary medicine to improve livestock health and increase yield^21–23^. Ecto-parasites force animals to expend a large amount of energy in defensive behavior, reducing feeding/grazing time, feed efficiency and milk production in the case of dairy cattle^24–26^. Endo-parasites alter nutrient absorption, which leads to slower weight gain, reduced milk production and lower carcass quality^27^. Among approved endectocides, the best-known classes are avermectins which include ivermectin, and several analogs such as moxidectin, selamectin and eprinomectin^23^.

Ivermectin has been widely used by veterinarians for the control of parasites of livestock and companion animals for decades^28^. It has low mammalian toxicity and is approved for human use^29^. Ivermectin binds selectively and with high affinity to glutamate-gated chloride ion channels in muscle and nerve cells of invertebrates, including malaria vectors^30^. This binding causes an increase in the permeability of the cell membrane to chloride ions and results in hyperpolarization of the cell, leading to paralysis and death^31^.

There is an opportunity to manage residual malaria transmission driven by partly zoophagic vectors by administering endectocides, such as ivermectin, to livestock^32–36^. Recent semi-field and field studies on *An. arabiensis* and *An. coluzzii* fed on ivermectin-treated cattle have found reduced survival, reduced blood meal digestion, reduced oviposition and reduced fecundity^34,35,37^. Although all these studies show a significant impact on important mosquito life-history traits that should decrease the overall vectorial capacity, direct evidence that malaria prevalence is affected is lacking.

### Livestock, malaria and endectocides, where to start?

Malaria elimination may not be attainable with scale-up of IRS and/or LLINs alone in areas dominated by partly zoophilic vectors such as *An. arabiensis*. Instead, efforts could be complemented with vector control measures that address mosquito behavior leading to residual transmission, such as larval source management and systemic application of endectocides in humans or livestock^35,38^. This review focuses on cattle given their large biomass, frequent rearing in rural communities and economic importance throughout Africa. Although coordinated mass-treatment of cattle might be logistically challenging, existing small-scale studies show significant impact on partly zoophilic vectors^35,39^.

Given the potential added value of endectocide-treated cattle to reduce malaria transmission and the need for evidence-based priority-setting in order to optimize vector control, this study aims to identify the regions of Africa where a high malaria burden coincides with high cattle densities in the presence of a problematic vector with important feeding plasticity: *Anopheles arabiensis*.

## Methods

The raw data on cattle density per squared kilometer was obtained from the International Livestock Research Institute (ILRI), Food and Agriculture Organization of the United Nations (FAO) and the Université Libre de Bruxelles (ULB-LUBIES) (https://livestock.geo-wiki.org/Application/index.php). For *Plasmodium* parasite rate among 2–10 years old and the distribution of *Anopheles arabiensis*, data aggregated and made public by the Malaria Atlas Project was used (https://map.ox.ac.uk/explorer/#/explorer) (downloaded October 2018).

The geographic attributes of the datasets (extents and projections) were standardized along with granularity to one surface using R (code at https://github.com/joebrew/cowsquitoes). The prevalence scale (0–1), the vector prevalence scale (0–0.847) and the cattle per square kilometer scale (0-infinite) were standardized to one 0–100 metric via percentilization (of Sub-Saharan African maximum).

Following standardization, the three percentilized metrics were combined by simple product. So, in the combined score, 0 means no *Plasmodium falciparum* among 2–10-year-olds, no *An. arabiensis* and no cattle, whereas 100 would theoretically mean the maximum (respective) values of the three. Anything between the two extremes represents some combination. This method assumes an equilinear value of percentilized *Plasmodium falciparum* parasite rate, *An. arabiensis* density and cattle density rate.

This combined scored was calculated by country and first administrative division which were then ranked by median score and proportional area above the continent’s mean.

## Results

**Figure 1:**
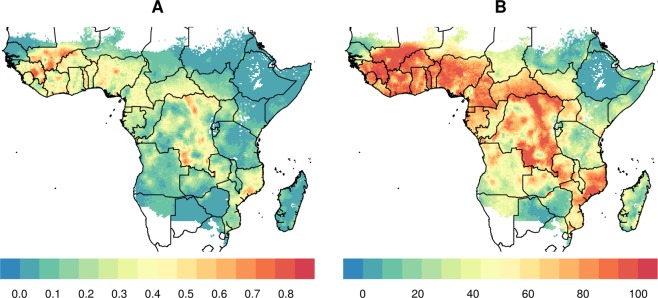
Plasmodium prevalence in 2–10 years old, using data aggregated and made public by the malaria Atlas Project (**A**) raw and (**B**) percentilized.

**Figure 2:**
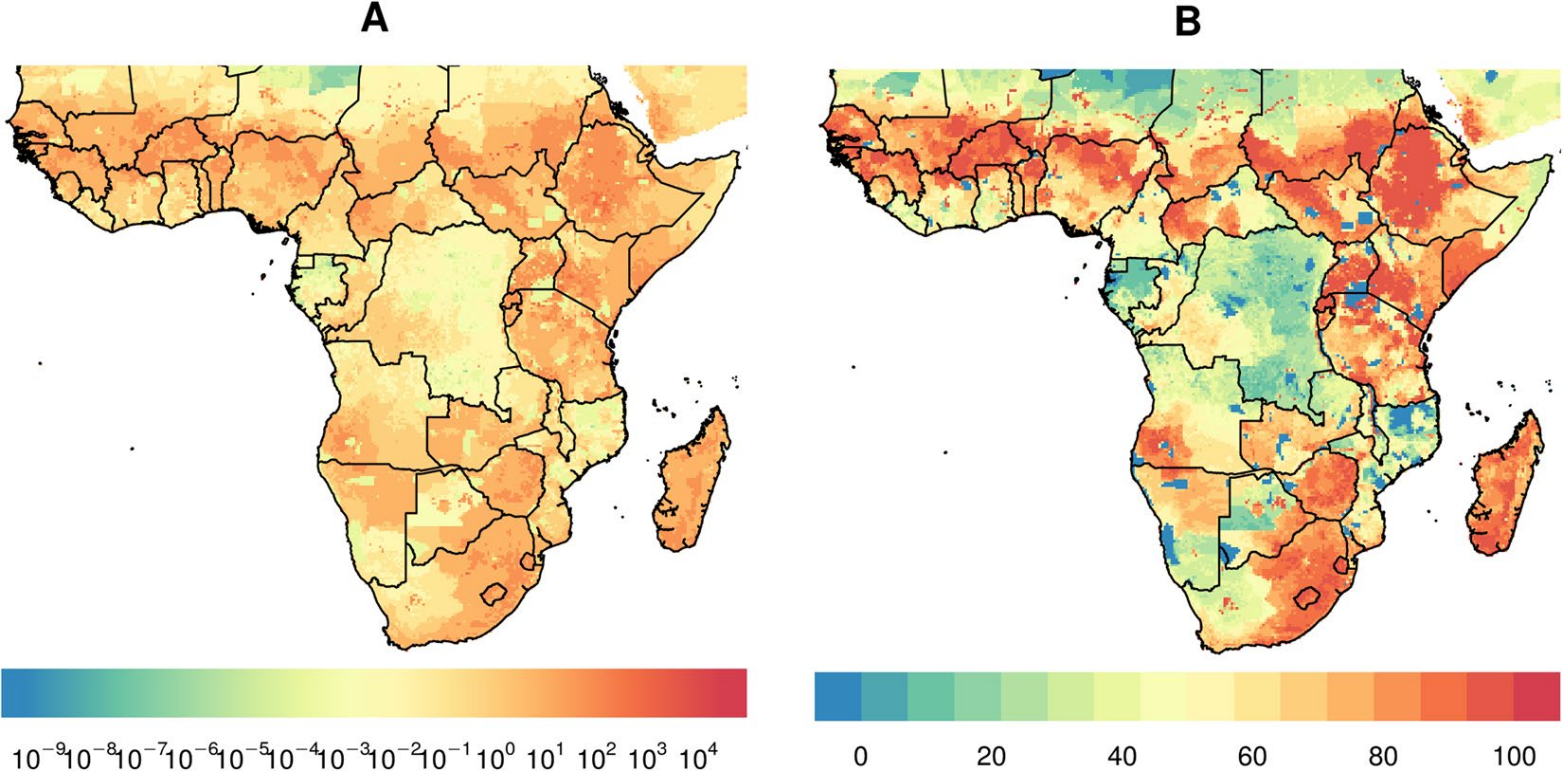
Cattle density per square kilometer (**A**) raw and (**B**) percentilized, obtained from the International Livestock Research Institute (ILRI), Food and Agriculture Organization of the United Nations (FAO) and the Université Libre de Bruxelles (ULB-LUBIES).

**Figure 3:**
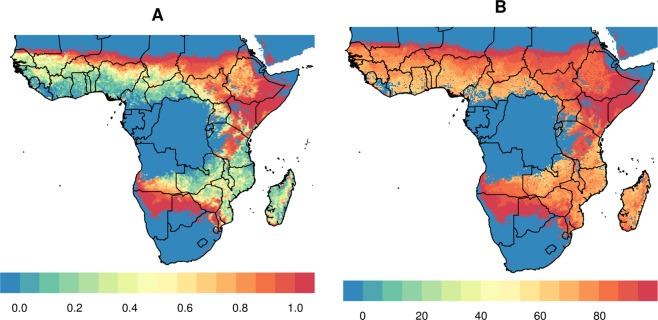
Density of Anopheles arabiensis (**A**) raw and (**B**) percentilized. Data aggregated and made public by the Malaria Atlas Project.

**Figure 4:**
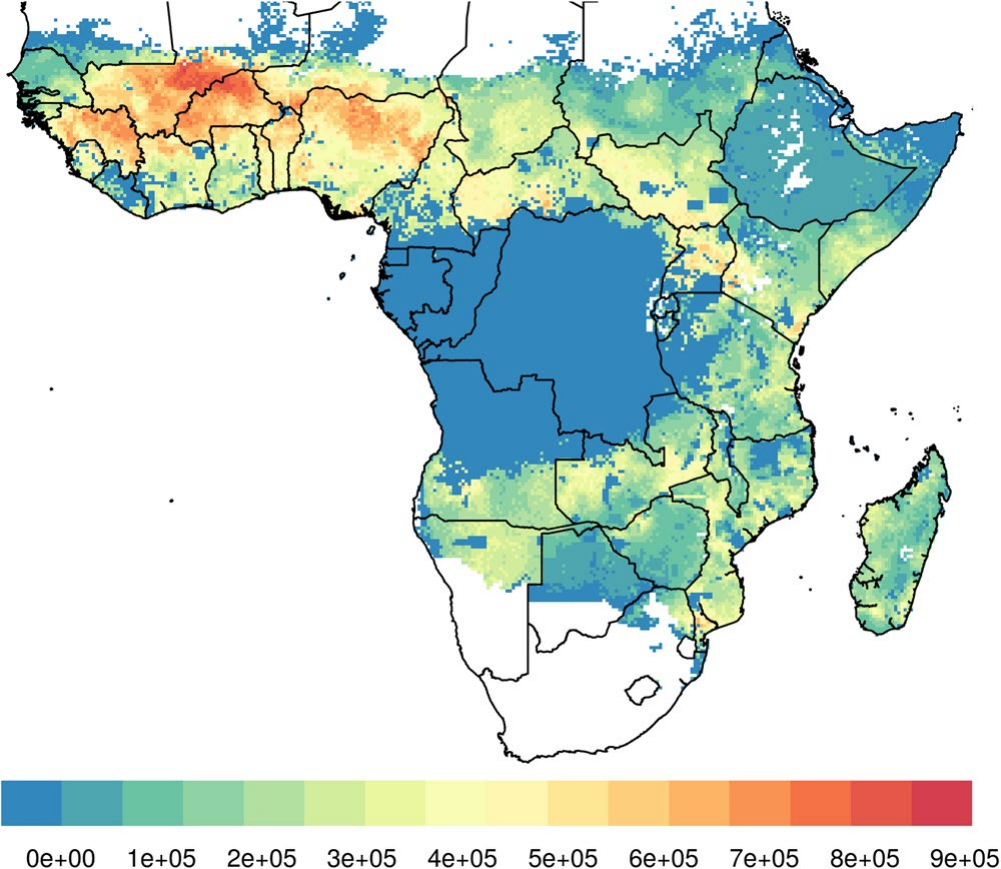
Mapping the results of the combined score obtained by combining the percentilized Plasmodium prevalence, and density of cattle and *Anopheles arabiensis*.

**Figure 5:**
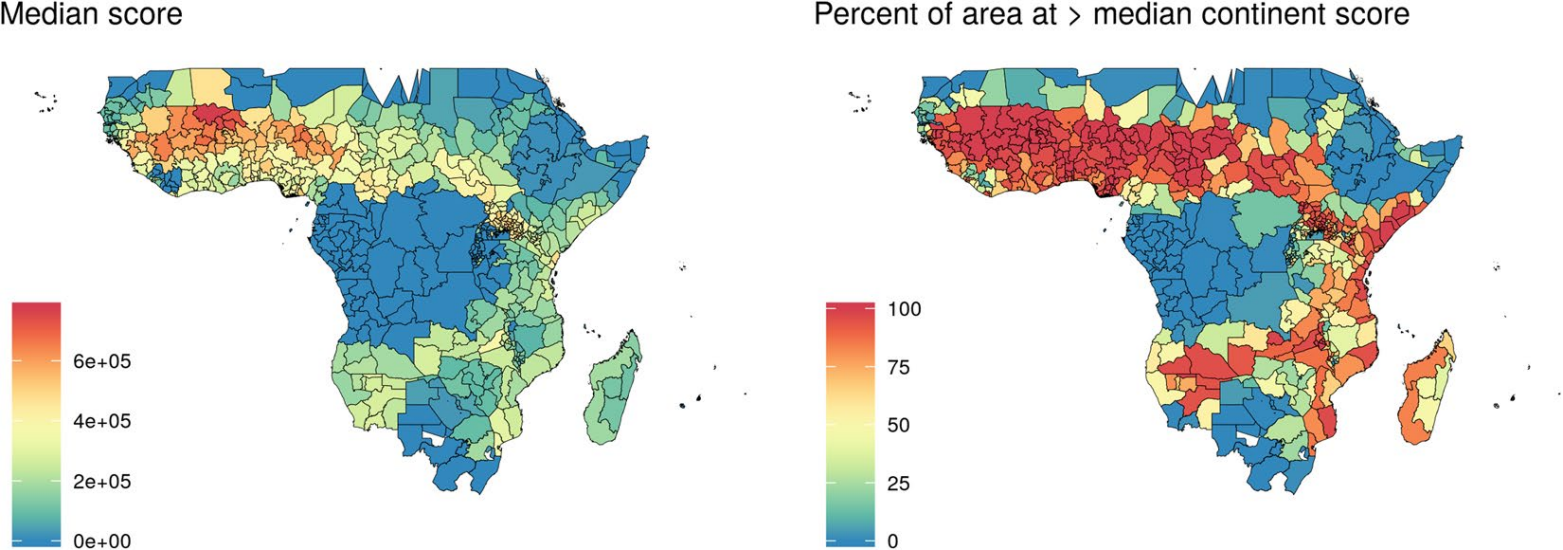
Mapping the combined score and ranking first administrative divisions by (**A**) median score in that unit and (**B**) divisions with part of their territory above the median continent score.

In Figs 1–5, maps of Africa are shown to investigate regions that could benefit to the largest extent from treating cattle with endectocide in order to reduce malaria transmission. As per Fig. 1, the greatest burden of malaria in Africa is observed in western and central Africa, as well as in regions of some southern African countries, including Mozambique, Malawi and Zambia. Figure 2 shows high cattle density in most non-deserted regions of Africa with the exception of large parts of some central African countries (Democratic Republic of Congo and Gabon), possibly due to the local dominance of rainforest, as well as large areas of Namibia, western South Africa and northern Mozambique. Presence and density of *An. arabiensis* are presented in Fig. 3. Noticeably, regions of high density overlap largely with regions of high cattle density (Figs 2B and 3B). In areas with high *An. Arabiensis* density (greater than the median pixel-specific value), the average percentilized cattle density is 70.1, compared with 58.2 in areas with no *An. Arabiensis* at all and 37.7 in areas with low (below median) *An. Arabiensis* density. By the same token, areas with high cattle density (greater than the median pixel-specific value), the average percentilized *An. Arabiensis* density is 51.2, relative to only 6.75 in areas with no cattle at all and 9.49 in areas with low (below median) cattle density.

In a similar fashion, there seems to be a relationship between parasite prevalence (Fig. 1) and cattle density (Fig. 2), albeit this relationship is less clean that the relationship between cattle and *An arabiensis*. Areas with parasite presence have more cattle than non-parasite areas. In areas with high parasite density (greater than the median pixel-specific value), the average percentilized cattle density was 54.4, compared with 37.3 in areas with no parasites at all. That said, cattle density was highest (67.7) in areas with low (below median) parasite density. By the same token, areas which are dense in cattle have more parasite than non-cattled areas. For areas with high cattle density (greater than the median pixel-specific value), the average percentilized parasite density is 45.4, much higher than 28.7 in areas with no cattle, but slightly lower than the 58.8 in areas with low (below median) cattle density.

Figure 4 shows the index combining disease burden, cattle density and *An. arabiensis* density, thus indicating the locations with the highest potential of benefitting from treating cattle with endectocide. These areas are widespread in most sub-Saharan western African countries; somewhat present in Central African Republic, Kenya and Uganda; and punctual in Mozambique, Angola and Madagascar. Chad, South Sudan, Zambia, northern Namibia and southern Somalia display medium scores quite uniformly across the territory. In all other malaria-endemic countries surfaces with low scores prevail, namely in Sudan, Djibouti, Eritrea, Ethiopia, Botswana, Malawi and central African countries, mostly attributable to the low local density of *An. arabiensis*.

When extrapolating overall surface results to the first administrative level, ranking them by suitability for endectocide-treated cattle according to their median score (Fig. 5A) and according to the percentage of unit area that is above the median continent score (Fig. 5B), the density of highlighted administrative units is most striking in a distinctive climatic block involving several regions of different countries: the savanna region underneath the Sahel in west Africa, where the potential for intervention is the greatest. There is, however, a scatter of administrative units highlighted as potential benefiters from the intervention in central and eastern sub-Saharan Africa, including parts of Somalia, Kenya, Uganda, Tanzania, Zambia, Malawi, Mozambique, Madagascar, Namibia and Angola. An interactive map with the individual components of the combined score in each first administrative division in Africa has been made available online at http://www.databrew.cc/cow.html.

## Discussion

Controlling residual malaria transmission needs a comprehensive approach, integrating interventions that target the various mosquito behaviors^11^ and using measures that go beyond personal protection^12^. The specific portion of residual transmission that is driven by partly zoophilic vectors could be controlled with endectocides that reduce the lifespan of mosquitoes feeding upon cattle^40^. This intervention appears to be a suitable vector control tool in vast areas of the African continent. This may be attributable to the fact that it targets *An Arabiensis* capitalizing on its partly zoophilic nature, together with other influencing factors. Recent evidence has identified *An Arabiensis* as the *Anopheles* species that is most widespread in the African continent^41^.

The analysis presented here identifies the African areas with theoretical higher potential to benefit from endectocide-treated cattle (ETC) as a complementary strategy for vector control. With no room for doubt, West Africa appears as the African region where ETC would be most effective. Not only does high under-10 malaria prevalence overlap with high cattle density, but it also coincides with the highest densities of the partly zoophagic *An. arabiensis* that currently sustain local residual transmission^11,12^. In four west African countries (Burkina Faso, Guinea, Benin and Togo) all first level administrative units present median combined scores that are half of the maximum score or above (i.e., median score ≥ 50%). Burkina Faso has the administrative units with the highest median scores across Africa, together with the Sahel and Sudan regions of Mali. This makes these four countries highly eligible for country-wide ETC, and together with the named areas of Mali a region-wide strategy seems highly appropriate. Currently, West Africa is the region with the highest under-10 malaria prevalence and the only region in Africa where seasonal malaria chemoprevention (SMC) is part of the standard of care for children under five years of age. The addition of ETC may be one of the necessary ingredients for malaria control and eventual elimination in a region where the latter possibility cannot be contemplated with currently available interventions alone.

Previous modelling studies have estimated that using a combination of currently existing interventions (LLINs, IRS and three rounds of mass drug administration, or MDA) pre-elimination levels would only be achievable in 26 (95% CI 22–29) of 41 African countries within the next 20 years^42^. In the remaining 15 countries, additional new interventions will be necessary to achieve pre-elimination levels. In Fig. 5, our analysis sheds light on the countries with administrative units that have the greatest potential of benefiting from ETC (median score ≥50%). All the countries highlighted in our study, with the exception of Sierra Leone and Guinea, are amongst those remaining 15 countries where previous models have predicted that no combinations of the other key malaria interventions would make it possible to achieve pre-elimination in the next 20 years^42^. Therefore, ETC could become a positive contribution to the malaria toolbox in many regions of Africa, and especially in regions of those countries where new interventions are most needed. Testing the effectiveness of ETC in combination with other strategies seems particularly relevant in the administrative units highlighted in these 13 countries.

Although country-wide ETC is predicted to be useful across an entire country in only a few examples (Fig. 5), its potential in certain administrative units is certainly worthy of exploration. Targeting malaria interventions at the local level has the potential to maximize impact and cost-effectiveness. For example, a recent study evaluating the cost-effectiveness of combinations of different malaria interventions (insecticide treated nets, IRS, MDA and SMC) across Africa estimates 32.1% (95% CI 29.6–34.5) cost savings when adopting policies at the provincial level compared to country-wide policies^42^. On the other hand, the potential for a region-wide intervention (i.e., the savannah region underneath the Sahel in West Africa) (Fig. 5) may provide the opportunity for leveraging economies of scale and transfer of knowledge and skills across countries to address the pressing issue of malaria control in West Africa and beyond.

Interestingly, upon observing the highly overlapping distributions of cattle and of *An. arabiensis* one is compelled to suspect a certain level of coevolution, more so given the zoophagic nature of the vector. Evidence of the coevolution of other members of the *Anopheles* complex, namely *An. gambiae* and its vertebrate host for bloodmeals (humans), has been previously observed and reviewed by Cohuet *et al*.^43^, reflecting the plasticity of the vector’s genetics and its genetic capacity to specialize to the human host and its environment. On the other hand, their co-existence could be coincidental or dependent upon factors such as requiring similar living conditions: both cattle and *An. arabiensis* may not thrive in deserted nor rainforest regions, which are almost the only areas of Africa where neither of them are densely present. Cattle go where people go. However, the presence of *An. funestus* and *An. gambiae* in the central African rainforest indicates the viability of the *Anopheles* mosquito in such environments.

Similarly, areas of higher malaria prevalence may also have a tendency towards higher cattle densities, albeit the overlap is less strict than in the case of *An. arabiensis* with cattle. This observation provides support for a zoopotentiation effect in some areas, where the presence of cattle as an alternative mosquito feeding source may be attracting an increasing number of mosquitoes towards human populations if they live close to cattle. In some regions and/or households, cattle are kept outdoors and away from the home hence posing no major risk of zoopotentiation, while in other regions cattle are kept indoors or in close proximity to humans due to security reasons. Although speculative at this point, the observation warrants further investigation, as documented zoopotentiation would further support ETC implementation, particularly in areas were cattle ownership and nocturnal husbandry close to the household are more frequent. Interestingly, the most cattle-dense areas are those with high parasite prevalence but not those with the highest parasite prevalence, and the most parasite-dense areas are those with some cattle but not those with most cattle. This observation may provide a hint towards further investigations on the effect of different husbandry practices, the vectors present in those areas, on a potential relationship between cattle and parasites by which both may thrive within a certain equilibrium between the species, or on any other potentially influencing factors.

For this exercise, the currently most complete and updated publicly available data were used. The three datasets used employ measurements at sub-country levels to model continent-wide estimates at quite granular levels of spatial resolution. A variety of data sources are inputted for the creation of those three models, and the accuracy of the original models in different regions depends on the nature, quality and granularity of data collected. Malaria prevalence and *Anopheles* data are based on an extensive database containing over 40,000 geo-referenced cluster locations’ parasite rate survey records^44^, and are therefore the probably most reliable models of the three. Most data used to produce the models are more recent than 2000^45–47^. Given that for each raster surface, the data inputted in our models is not field data but modelled data, the parameters obtained are also somewhat speculative. To this extent, the maps presented here should be understood as the first step towards obtaining more insight on the areas where it is most relevant to conduct more targeted investigations (including obtaining field data on malaria prevalence, cattle density and *An Arabiensis* density) and test ETC in different contexts.

Amongst the limitations of this current work stands the lack of data on the proportion of the malaria prevalence that is attributable to transmission via each mosquito species in each surface. This limits the ability to infer whether ETC, an intervention targeting only mosquitoes that display plasticity in the species they feed upon, would be able to avert most malaria transmission or only a small part of it in areas presenting a wealth of mosquito species. The same applies to the lack of granular data on mosquito behaviors and other factors that potentially affect the effectiveness of the intervention, including differing vectorial capacities of the same mosquito species across Africa^43^ and the diversity of cattle ownership and husbandry practices that determine proximity of cattle to humans.

Recognizing these limitations, the maps we have generated employ percentilization of the parameters imputed to show the relative potential for an ETC intervention compared to the rest of areas in Africa. Therefore, we have effectively identified ideal locations where details on vector contribution to proportion of disease transmission, mosquito feeding behaviors and cattle husbandry practices should be investigated to further improve our understanding of where ETC may be most effective.

If ETC was proven effective for reducing malaria, other gaps in knowledge would need to be addressed. Some examples include evaluation of ETC cost effectiveness in combination with other strategies, assessing the intervals of reapplication of endectocides to ensure lengthy efficacy, assessing the best preemptive approach to resistance (mosaic, rotation, refugia or alternation) through operational research and finally evaluating the attitudes and perception of cattle owners to the proposed strategy, particularly if human ivermectin is being piloted or deployed in the same areas.

It is particularly important to consider the cattle owners’ perception of ETC, as endectocides have long been used for control of both ecto- and endo-parasites that reduce productivity (e.g., low efficiency of feed conversion, weight gain and milk production) and cause economic losses^21,22,48^. Treatment of livestock with endectocides has also been successful against *Tse tse* flies in sub-Saharan Africa^49^, tick-borne diseases worldwide, and a variety of other biting and/or nuisance insects including flies, biting midges, mites and lice^22^. At approved labeled doses, topically or systemically applied endectocides provide the sufficient and/or sustained dosage required for reduction of mosquito survival rate below elimination threshold^50,51^. Consequently, by employing endectocides for malaria control, cattle owners will also enjoy direct and indirect benefits such as enhanced milk production, reduced burden of parasites and healthier animals^52^.

Being able to capture the potential direct and indirect benefits associated with ETC would be critical in any cost effectiveness analysis of the intervention. In addition to the potential economic gains achieved from improved livestock productivity, healthier livestock have a profound positive effect on human nutrition and health, especially in poor communities highly reliant on livestock^53^. Livestock parasites contribute to human malnutrition by affecting both the food available to human populations and the income generated by populations dependent on livestock production. These two factors in turn contribute to poverty and a weaker physical state, making populations more vulnerable to disease and malnutrition, and less able to obtain protection. Recent mathematical modelling suggests that non-zoonotic livestock disease can ultimately have a more significant impact on human health than human disease alone^53^, making the treatment of livestock with endectocides very appealing.

In conclusion, killing competent mosquito vectors of malaria when they feed upon animals by treating livestock with endectocides may be critical for tackling residual transmission driven by zoophagic and opportunistic mosquitoes^40^. Through this study, we have identified those geographical areas where using an ETC intervention would be most likely to prove effective at reducing cases of malaria, and where we should focus our research efforts going forward. As an additional, and perhaps not insignificant benefit, ETC has the potential to improve the overall health and nutrition of human populations through its impact on livestock health. Furthermore, because endectocides’ mode of action differs from that of pyrethroids, they provide a novel complementary intervention to LLINs and IRS. In essence, employing ETC interventions could reduce the burden of insecticide resistance and prolong the effectiveness of other insecticide-based interventions. This is most necessary in the context of addressing *An arabiensis*, the main vector targeted through this intervention, as it is the only *Anopheles* species that has been reported to display resistance to the four main types of insecticide^41^. Should ETC become a new intervention in the toolbox for malaria control and elimination, the current analysis shows that those areas where it has the potential to be most effective are also those that most need it, where elimination is not within reach with the currently available interventions – perhaps not coincidentally.

## Data Availability

All datasets are publicly available from the cited sources. All code is available at https://github.com/joebrew/cowsquitoes. The interactive online map is publicly available at http://www.databrew.cc/cow.html.
